# Stevens-Johnson Syndrome

**Published:** 2016-12-07

**Authors:** Jake Laun, Katie Laun, Mayssan Muftah, Amanda Zimmerman, Joshua B. Elston, David J. Smith

**Affiliations:** ^a^Division of Plastic Surgery, Department of Surgery, University of South Florida Morsani College of Medicine, Tampa; ^b^Department of Emergency Medicine, Florida Hospital, Orlando

**Keywords:** Stevens-Johnson syndrome, SJS, toxic epidermal necroysis, TEN, rash

## DESCRIPTION

A 45-year-old African American HIV-positive man presented with a 6-day history of worsening rash that began on his trunk and progressed to involve his extremities, oral mucosa, and urethral meatus. He reported a recent history of starting sulfamethoxazole-trimethoprim for pneumocystis prophylaxis.

## QUESTIONS

1. **What is Stevens-Johnson syndrome (SJS) and how is it classified?**
2. **What causes SJS and how does it present?**
3. **How is SJS treated?**
4. **What is the prognosis of SJS?**

## DISCUSSION

Stevens-Johnson syndrome is a severe and potentially lethal disease due to an immune-complex–mediated hypersensitivity reaction involving the mucous membranes and skin.^1^ First described in 1922, SJS was originally thought to be a variant of erythema multiforme (EM); however, this has fallen out of favor, as SJS is mainly due to a drug reaction whereas EM is mainly a parainfectious process.^2^ SJS occurs on a spectrum based upon total body surface area (TBSA) of skin affected, with the most severe form referred to as toxic epidermal necrolysis (TEN), or Lyell's syndrome. SJS encompasses less than 10% TBSA; SJS/TEN overlap occurs between 10% and 30% TBSA, and TEN encompasses more than 30% TBSA.^2^

**Figure 1:**
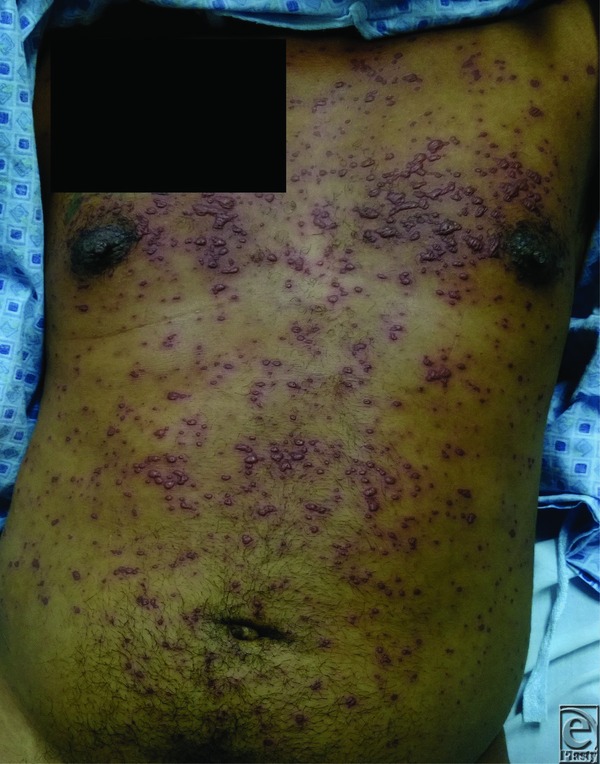
Anteroposterior view of the trunk with coalescing vesicobullous lesions

**Figure 2:**
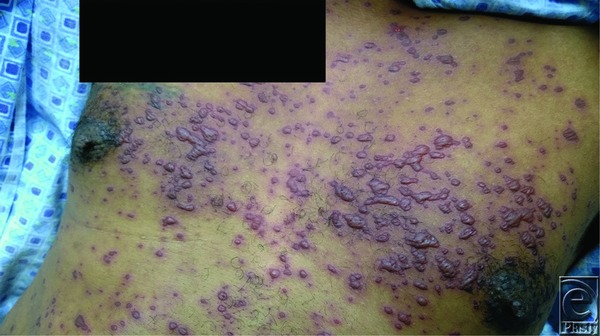
Closer view of the trunk, as shown in Figure 1, highlighting the confluent nature of the left chest lesions. These lesions demonstrated a positive Nikolsky sign, indicating a plane created via shearing force displacing the epidermis.

**Figure 3:**
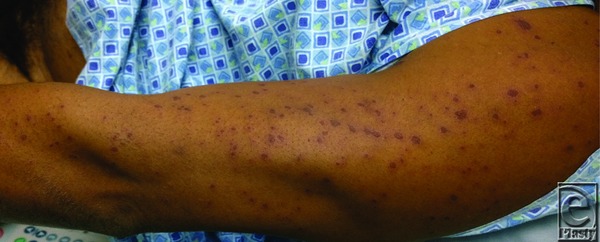
Lesions beginning to form on the left upper extremity have yet to become fluid-filled.

SJS/TEN can occur in any individual but are more common in children and the elderly.^3^ Although there have been cases reported after a viral illness (mainly herpes simplex) or mycoplasma infections, more than 90% of cases are medication induced—the main culprits being antibiotics (sulfonamides and β-lactams), nonsteroidal anti-inflammatory drugs, and antiepileptics (phenytoin and carbamazepine).^3^,^4^ Initial presentation can be nonspecific, with fever, malaise, cough, sore throat, or eye discomfort all appearing before the cutaneous manifestations. Following this prodrome is the onset of mucosal ulcerations and then progression to cutaneous, dusky red vesiculobullous-appearing atypical target-like lesions on the trunk and face that evolve over the course of 2 to 15 days (Figs 1-3).^3^,^5^ These cutaneous manifestations have a positive Nikolsky sign due to dermal-epidermal junction involvement (Fig 2).^6^ If skin lesions are seen, biopsy is warranted to assist with diagnostic workup. The classic pathology findings are dermal monocyte infiltrate with full-thickness epidermal necrosis.^1^,^3^ Eye involvement can range from erythema and eyelid edema to corneal ulcerations and can be debilitating in the long term.^1^,^3^,^5^

The initial step in management should be discontinuation of medication suspected of precipitating the event—otherwise care is largely supportive. Patency of the airway, hemodynamic stability, and overall fluid status must continually be assessed. There should be a low threshold for enteral feeding tubes to provide the necessary calories for wound healing if oral ulcerations prevent adequate intake.^1^ Urinary catheters may be useful if genital involvement is present. In regard to cutaneous involvement, care is directed toward preventing shear forces that would further denude skin. Nonadherent silver-containing dressings can be used to cover all affected areas and minimize bacterial colonization of skin as well as reducing the amount of dressing changes that may further contribute to skin sloughing.^7^ Systemic therapy such as corticosteroids, intravenous immunoglobulin, prophylactic antimicrobials, and plasmapheresis is controversial, with no controlled study showing a clear benefit on mortality or reducing disease progression.^3^ Involved skin should not be excised or debrided. Consultations with subspecialists such a Critical Care, Ophthalmology, Urology, and Infectious Diseases should be considered.

Disease prognosis is variable, with the risk of mortality increasing with the severity of the syndrome. SJS has a mortality of approximately 5% and TEN has the worst mortality of approximately 16% to 55%.^3^,^6^,^8^ Scoring systems, such as the SCORTEN, exist to assess the risk of mortality, with factors including patient age, TBSA involved, concomitant malignancy, as well as several initial serum chemistries.^8^

Immune-complex–mediated hypersensitivity reactions such as SJS/TEN can be devastating. Initial care should include discontinuation of the offending medication. Further management is supportive and aims to prevent secondary complications, hypovolemia, and infection. Wound care is provided with silver-containing dressings and preventing further shearing of skin. Patients should be monitored closely in an intensive care setting, with high suspicion for development of pulmonary or infectious processes, as these are the overwhelming cause of mortality.
